# Microbial Communities in the Upper Respiratory Tract of Patients with Asthma and Chronic Obstructive Pulmonary Disease

**DOI:** 10.1371/journal.pone.0109710

**Published:** 2014-10-16

**Authors:** HeeKuk Park, Jong Wook Shin, Sang-Gue Park, Wonyong Kim

**Affiliations:** 1 Department of Microbiology, College of Medicine, Chung-Ang University, Seoul, South Korea; 2 Division of Pulmonology and Allergology, Department of Internal Medicine, College of Medicine, Chung-Ang University, Seoul, South Korea; 3 Department of Applied Statistics, Faculty of Business and Economics, Chung-Ang University, Seoul, South Korea; National Jewish Health, United States of America

## Abstract

Respiratory infections are well-known triggers of chronic respiratory diseases. Recently, culture-independent tools have indicated that lower airway microbiota may contribute to pathophysiologic processes associated with asthma and chronic obstructive pulmonary disease (COPD). However, the relationship between upper airway microbiota and chronic respiratory diseases remains unclear. This study was undertaken to define differences of microbiota in the oropharynx of asthma and COPD patients relative to those in healthy individuals. To account for the qualitative and quantitative diversity of the 16S rRNA gene in the oropharynx, the microbiomes of 18 asthma patients, 17 COPD patients, and 12 normal individuals were assessed using a high-throughput next-generation sequencing analysis. In the 259,572 total sequence reads, α and β diversity measurements and a generalized linear model revealed that the oropharynx microbiota are diverse, but no significant differences were observed between asthma and COPD patients. *Pseudomonas* spp. of Proteobacteria and *Lactobacillus* spp. of Firmicutes were highly abundant in asthma and COPD. By contrast, *Streptococcus*, *Veillonella*, *Prevotella*, and *Neisseria* of Bacteroidetes dominated in the healthy oropharynx. These findings are consistent with previous studies conducted in the lower airways and suggest that oropharyngeal airway microbiota are important for understanding the relationships between the various parts of the respiratory tract with regard to bacterial colonization and comprehensive assessment of asthma and COPD.

## Introduction

Asthma and chronic obstructive pulmonary disease (COPD) are common chronic inflammatory diseases of the airways, and their prevalence and mortality rates continue to increase, establishing them as leading causes of disability worldwide [1]. The World Health Organization (WHO) estimates that 235 million people are annually affected by asthma and approximately 250,000 people die per year worldwide [2]. COPD also affects 329 million people worldwide, resulting in approximately 3 million deaths annually [3], [4].

Clinical studies of chronic respiratory diseases have been focused on their association with common respiratory viruses. Respiratory viral infections are common and usually self-limiting illnesses in healthy adults but are a major cause of exacerbations in patients with asthma and COPD [5]. One of the triggers for development of asthma and COPD is impaired lung function, caused due to bacterial infections. In asthma, *Streptococcus pneumoniae*, *Haemophilus influenzae*, and *Moraxella catarrhalis* are clinically relevant contributors to asthma exacerbations, particularly when sinusitis is present [1], [2]. Atypical bacteria such as *Chlamydophila pneumoniae* and *Mycoplasma pneumoniae* may also be associated with asthma [6]–[8]. The role that bacterial pathogens play in acute exacerbations of COPD is controversial. *Pseudomonas aeruginosa*, *Haemophilus influenzae*, *Moraxella catarrhalis*, and *Streptococcus pneumoniae* are known to be associated with a significantly increased risk of exacerbation of COPD. However, the prevalence of bacterial pathogens in the lower airways was found to be the same during acute exacerbations and stable disease; thus, changes in bacterial load are unlikely to be an important mechanism underlying exacerbation of COPD [9]–[11].

Human microbiota is diverse across individuals and different sites in the body. Oral microbiota are known to be involved in upper and lower respiratory infections, which can also develop into atopic airway diseases such as allergic rhinitis and bronchial asthma [12]. The oropharynx is constantly exposed to both inhaled and ingested microbes, including those cleared by mucociliary mechanisms from the upper and lower respiratory tracts and those contained in saliva [13]. The clinical relevance of the microbiota of the oropharyngeal wall is unknown. However, it is known that the diversity of the oral microbiota can be affected by antibiotics, probiotics, diet, and gut microbiota [14].

Oral microbiota can trigger and strike as a critical component in conditions like Bronchial asthma and COPD [15], particularly considering that the normal healthy lung is not a sterile organ as previously assumed [16]. To date, the relationship between airway microbiota and chronic respiratory diseases has been investigated only in lower airway samples such as sputum and bronchoalveolar lavage (BAL) samples [17], [18], [19]. However, the microbial inhabitants of the upper respiratory tract in asthma and COPD patients has been insufficiently characterized. The induction or suppression of systemic immune tolerance to challenge antigens can be attributed to the link between oral tolerance and airway tolerance. Although strong evidence exists to implicate bacterial infections in the course and pathogenesis of airway diseases, systematic studies of organisms in the airways are lacking [12]. So it becomes crucial to define the characteristics of the oropharyngeal microbiota, unfolding the pathogenic roles of these microorganisms. This in turn can lead to identification of potential targets and the discovery of novel therapies for the prevention and treatment of oral complications [20].

Traditionally identification of bacteria in the oral cavity has been done by culturing methods. Analysis of microbiota through culture-dependent methodologies is both expensive and time-consuming in comparison with many of the available culture-independent methods. It is evident to assess minor or non-dominant populations, culture-independent strategies are important for analysis of microbial ecology. Previous studies demonstrate culture-independent approaches have revealed more diverse microbiota compared to culture-dependent microbiota, of which nearly 40–60% bacterial taxa in the mouth were uncultured or validly described [21].

Recent high throughput methods like next-generation sequencing have revolutionized the way diversity of microbial communities in varied environments, including the human body is analyzed [22]. In this study, we aimed to comprehensively characterize the bacterial communities of the oropharyngeal airway in asthma and COPD patients relative to healthy individuals using a high-throughput 16S rRNA gene-pyrosequencing analysis.

## Methods

### Study subjects

All of the samples were collected from individuals with newly diagnosed asthma and COPD in accordance with the ethical guidelines mandated by the commission a protocol (number #12-0015) that was approved by the University’s Human Subjects Institutional Review Board (IRB) of Chung-Ang University College of Medicine, Seoul, Korea. Human subjects remain anonymous and the informed consent was written. All participants gave written informed consent for participation in the study. The current diagnosis of asthma was based on a history of wheezing, shortness of breath, and cough, which were variable in severity and frequency [23], [24]. COPD patients were further classified into four distinct stages according to Global Initiative on Obstructive Lung Disease (GOLD) guidelines [25]. Patients with bronchial asthma had mild intermittent disease and were treated as being naive. For the oropharyngeal swabs, 3M quick swabs (3M Microbiology Products; St. Paul, MN, USA) were inserted into the oral cavity until resistance was met at the oropharynx. The swabs were then rotated 180 degrees and withdrawn while taking care not to contaminate the swabs with oral bacteria. After swabbing, the swabs were transported immediately to the lab for identification and subsequent microbiomic analysis. Each swab sample was aseptically placed in a microtube and centrifuged at 14,000 rpm for 10 min, and the supernatant was removed. To detect possible contamination, negative controls were prepared and subjected to the same procedures.

### DNA extraction from oropharyngeal swab samples

The first step in the analysis was extraction of DNA from the bacterial pellet that was obtained from oropharyngeal swab samples, followed by polymerase chain reaction (PCR) analysis. Samples were extracted individually by using the cetyltrimethylammonium bromide method [26]. Purified DNA was dissolved in sterile water with 40 µg/mL RNase A and quantified using an Infinite 200 NanoQuant microplate reader (Tecan; Männedorf, Switzerland) at a wavelength of 260 nm.

### GS-FLX 454 pyrosequencing by using 16S rRNA gene amplification

PCR amplification of the 16S rRNA gene was performed following established procedures [27]. The V1–V3 region of the 16S rRNA gene was amplified with primers 8F (5′-CTGCTGCCTYCCGTA-3′) and 530R (5′-GTATTACCGCGGCTGCTG-3′) with the MID sequence. PCR was performed in final reaction mixtures of 25 µL containing 5–25 ng of genomic DNA, each primer at 0.4 mM, 0.2 mM dNTPs (Takara Bio; Shiga, Japan), 1.5 mM MgCl_2_ (Invitrogen; Carlsbad, CA, USA), 2.0 U of Hot Start Taq polymerase (Takara), and 1.0 µL of reaction buffer (Invitrogen). PCR amplification was performed on a GeneAmp PCR system 9700 thermocycler (Applied Biosystems; Foster City, CA, USA) under the following conditions: initial denaturation for 5 min at 94°C, followed by 30 cycles of 30 s at 94°C, 30 s at 55°C, and 1 min at 72°C, with a final extension for 10 min at 72°C and cooling to 4°C. The PCR products were resolved on a 1.2% Seakem LE agarose gel (FMC Bioproducts; Rockland, ME, USA) and visualized after ethidium bromide staining. 454 sequencing was carried out on the pooled PCR products of equal amounts. Amplicon pyrosequencing was performed using a 454/Roche GS-FLX Titanium instrument (Roche Molecular Systems; Branchburg, NJ, USA). After sequencing, the sequence file (.fna) and quality file (.qual) were recovered by MID groups. Quality control of the post-processing sequences was performed to eliminate sequences with ambiguous base calls, homopolymers >6, quality scores <Q25, read lengths <250 bp, and low-quality sequence ends and those without a 100% match to barcodes. Random sampling was used to analyze the diversity of the low reads of the sequence and the high reads of the sequence set. Tests of the sequence analysis methods are described in PRINSEQ [28]. The raw data were submitted to the National Center for Biotechnology Information-Sequence Read Archive (NCBI-SRA; http://www.ncbi.nlm.nih.gov/sra) with accession number SRX316595.

### 16S rRNA gene sequence analysis

The nucleotide sequence homology of the amplified 16S rRNA gene V1–V3 regions was determined using the nucleotide BLAST (BLASTn) program, which is available at the SILVA website (http://www.arb-silva.de/, Release 115) and the BLAST webpage of the NCBI. Accurate identification of organisms through comparative analysis of 16S rRNA gene sequences is strongly dependent on the quality of the database that is used. The curated Ribosomal Database Project (RDP; http://rdp.cme.msu.edu, Release10, update 32) provides bacterial and archaeal small subunit rRNA gene sequences in an aligned and annotated format, and has achieved major improvements in the detection of sequence anomalies [29]. Among all of the online tools provided by the RDP website, the RDP pyrosequencing pipeline includes information of Shannon-Wiener, Chao1, and ACE and operational taxonomic units (OTUs) through the rarefaction analysis tool. A phylogenetic tree was built using the neighbor-joining method in Clustal_X [30] with 1,000 bootstrap replicates. Fast UniFrac was used to evaluate the relatedness of samples [31], [32] (http://unifrac.colorado.edu/static/welcome.html). Weighted and normalized PCoA and Jackknife Environment Clusters were used to evaluate similarity among samples, where each sample represented an environment. Hierarchical cluster analysis was performed using complete linkage and UniFrac distances as a measure of similarity [33]. Weighted and normalized Principal Coordinate Analysis (PCoA) was performed to evaluate similarity among the samples, where each sample represented an environment. R software (version 2.15.2) with gplots and the RColorBrewer package was used for all other analytical processes.

### Statistical analysis

Factor analysis was applied to track the important variables from an observed set of variables which are influencing the final response. Principal components were identified with over 70% of the cumulative variance. The component matrix of the chosen principal components was calculated, and variables whose factor loadings lower than 0.6 were deleted. A generalized linear model (GLM) was constructed using the final set of chosen variables. The following equation (a) represents the GLM:
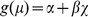
(a)where *t* is the total number of occurrences, 
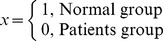
_,_ and 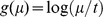 in the pooled group of samples from asthmatic patients, COPD patients, and healthy controls.

The parameter beta was tested at a 5% significance level by using (a).

## Results

### Study subjects

The 47 characteristics of the subjects are summarized in Table 1. For the current microbiota study, subjects were segregated into three groups: 18 patients with asthma (10 males, mean age 53.4±17.1 years; 8 females, mean age 55.0±13.0 years). All patients had a history of wheezing, cough, and dyspnea, with predicted post-bronchodilator FEV1/FVC 75.4±6.0, FEV1% 108.1±25.6, and FVC% 105.4±23.7. COPD subjects (15 males, mean age 68.9±7 years; 22 females, mean age 74.5±0.7 years) received a diagnosis of stage I (n = 6), stage II (n = 10), stage III (n = 1), or stage IV (n = 0) based on Global Initiative on Obstructive Lung Disease (GOLD) guidelines [25], with predicted post-bronchodilator FEV1/FVC 60.3±13.3, FEV1% 78.2±26.0, and FVC% 105.4±23.7. Healthy control subjects (six males, mean age 50.2±22.6 years; six females, mean age 59.3±17.7 years) exhibited no evidence of underlying respiratory disease.

**Table 1:** Characteristics of 47 subjects in the asthma, COPD, and healthy control groups.

	Asthma patients	COPD patients	Healthy controls
No. of subjects	18	17	12
Gender	M (10), F (8)	M (15), F (2)	M (6), F (6)
Average age (range)	53.4±17.1/55.0±13.0	68.9±7.2/74.5±0.7	-
FEV1%	108.1±25.6	78.2±26.0	-
FVC %	105.4±23.7	90.3±23.6	-
FEV1/FVC	75.4±6.0	60.3±13.3	-
Gold stage	-	I 6	-
	-	II 10	-
	-	III 1	-
	-	IV 0	-
Wheezing	18	-	-
Cough	18	-	-
Dyspnea	18	-	-

FEV1, forced expiratory volume in 1 s; FVC, functional vital capacity.

Date for FEV1% and FVC % are expressed as mean 6 SD.

### Sequence reads of the 16S rRNA gene

The diversity of microbiota in the oropharyngeal swab sample was characterized by sequencing the V1–V3 region of the 16S rRNA gene, which was amplified from genomic DNA samples of 18 asthmatic patients, 17 COPD patients, and 12 healthy controls. In total, 259,572 bacterial sequence reads were identified for three group samples with average read lengths of 273–476 bp (mean ± standard deviation, 398.9±41.9) (Table 2). The 191,616 sequence reads obtained after trimming of low-quality sequences were then analyzed (mean ± standard deviation, control: 3214±1136; asthma: 4140±3723; and COPD: 4619±5710).

**Table 2:** Characteristics of the study participants and sequencing results.

No.	Samplename	Sex	Age	Trimmedsequencesreads	OTUs	Avg. readlength	Chao1	Shannonindex
1	Normal 1	Male	32	3768	410	431.4	503.0	4.8
2	Normal 2	Male	74	3254	159	397.6	229.7	3.1
3	Normal 3	Male	71	1189	132	273.6	177.2	3.2
4	Normal 4	Male	67	3610	195	423.6	270.7	3.2
5	Normal 5	Male	30	634	77	313.3	113.3	3.2
6	Normal 7	Male	27	3608	280	437.7	384.1	4.2
7	Normal 8	Female	79	3868	203	403.9	245.4	3.3
8	Normal 10	Female	61	3857	232	418.5	303.0	3.9
9	Normal 11	Female	53	3803	327	398.7	477.1	3.9
10	Normal 14	Female	71	4286	242	406.5	292.7	3.8
11	Normal 15	Female	64	3832	81	410.4	87.2	2.0
12	Normal 16	Female	28	2864	144	416.4	198.1	3.6
	Normal average			3214±1136	207±99	394±49		3.5±0.7
13	Asthma 17	Male	55	3897	32	417.4	37.0	1.1
14	Asthma 18	Male	55	3251	165	407.0	224.9	3.6
15	Asthma 19	Male	58	3598	150	421.8	187.6	2.5
16	Asthma 20	Male	57	3115	29	476.7	32.5	0.3
17	Asthma 21	Male	28	8046	214	419.8	254.5	3.3
18	Asthma 22	Male	23	1509	105	390.6	165.0	3.0
19	Asthma 23	Male	69	2897	120	421.2	151.5	3.1
20	Asthma 24	Male	62	2075	155	402.8	313.0	2.8
21	Asthma 25	Male	74	2341	169	378.3	218.9	3.2
22	Asthma 26	Female	74	4063	78	433.3	88.1	1.3
23	Asthma 27	Female	51	1160	126	341.7	171.0	2.7
24	Asthma 28	Female	70	1924	41	451.8	54.0	1.4
25	Asthma 29	Female	51	4070	72	415.3	106.4	0.8
26	Asthma 30	Female	47	3821	211	425.9	311.6	3.8
27	Asthma 31	Female	30	890	29	346.6	45.5	1.8
28	Asthma 32	Female	55	2693	131	431.1	170.7	3.2
29	Asthma 83	Female	55	16657	189	420.1	213.8	2.5
30	Asthma 84	Female	62	8509	286	378.6	371.2	3.2
	Asthma average			4140±3723	128±73	410±89		2.4±1
31	COPD 85	Male	77	2914	190	411.9	245.1	3.7
32	COPD 86	Male	69	10568	297	340.8	372.0	3.8
33	COPD 87	Male	73	4214	157	390.2	218.9	3.0
34	COPD 88	Male	62	2563	75	420.1	110.8	1.5
35	COPD 89	Female	74	2998	124	391.7	159.3	3.2
36	COPD 90	Male	66	471	46	333.9	61.1	2.6
37	COPD 91	Male	73	24809	363	434.1	452.5	2.0
38	COPD 92	Female	75	2505	83	376.8	108.1	2.7
39	COPD 93	Male	57	3485	46	436.8	51.6	1.4
40	COPD 94	Male	68	3942	115	463.3	128.8	2.1
41	COPD 95	Male	76	2506	186	322.5	261.9	3.8
42	COPD 96	Male	77	4384	281	387.5	354.8	4.0
43	COPD 97	Male	75	1044	201	326.1	274.6	4.3
44	COPD 99	Male	66	2116	97	397.9	122.8	2.6
45	COPD 100	Male	72	6678	280	393.5	352.0	3.8
46	COPD 101	Male	53	1481	21	346.2	23.0	1.1
47	COPD 102	Male	69	1849	108	346.0	147.5	3.0
	COPD average			4619±5710	157±100	384±83		2.9±1
	Total reads			191,616	159±94			

The Chao1 estimator of total species richness sequences in healthy controls and asthmatic and COPD patients is described in Table 2. Ecological diversity was also estimated by using the Shannon–Wiener index, which takes into account relative abundance. This index is widely applied in ecological studies as a measurement of biodiversity, as it accounts for both the number of different taxa and their relative abundance. For this purpose, the 16S rRNA fragments were identified from the normalized pyrosequencing datasets and classified using the RDP classifier. Only 16S rRNA sequences of genus level with at least 80% confidence was used for Shannon-Wiener index. The resulting Shannon-Wiener index value demonstrated that the normal group was more diverse than the COPD and asthmatic groups because the sequence data obtained from the 454/Roche GS FLX Titanium sequencer provided a high estimate of biodiversity.

Species richness, diversity, and rarefaction calculations were used to analyze whether the diversity of the oropharyngeal microbial community was sufficiently covered by the sequence data. Species coverage, richness, evenness, and diversity were estimated for the combined data set for each of the bacterial sequences from the oropharyngeal samples. In addition, OTUs (5% difference) were calculated for the healthy control group and patients with asthma and COPD. Rarefaction analyses with RDP analysis curves at the OTUs reached saturation for the subjects with lower microbial diversity when the sequences of each sample type and the total number of OTUs were estimated. The diversity ranged from 29 to 410 OTUs with 95% similarity in each of the 47 samples (mean ± standard deviation, control: 207±99; asthma: 128±73; and COPD: 157±100) (Table 2). The calculation of rarefaction curves was used for all the samples, which showed that additional sampling would not have provided any additional OTUs (Figure S1) with very few exceptions. The samples from the asthma, COPD, and healthy control groups contained a total of 190 different bacterial genera representing 12 different bacterial phyla (Table S1).

### Phylum identification of bacterial communities

Seven bacterial phyla were detected, but most sequences were assigned to three phyla: Firmicutes, Proteobacteria, and Bacteroidetes. In 17 of 18 asthmatic patients, the majority of bacteria belonged to Firmicutes (59.8%), at a proportion higher than that in the healthy controls (48.4%). In addition, in 17 of 17 COPD patients, 61.6% of the bacterial phyla belonged to Firmicutes. Therefore, an increase in the prevalence of Firmicutes from 59.8% in the asthma sample to 61.6% in the COPD sample was observed. In the 12 healthy control samples, Proteobacteria and Bacteroidetes composed 29.02% and 16.20% of the bacteria community, respectively. In asthmatic and COPD patients, Proteobacteria accounted for 35.61% and 28.04% of the bacteria community, respectively. However, the percentage of Bacteroidetes was drastically decreased in the asthma (2.62%) and COPD (7.17%) groups (Table 3).

**Table 3:** Abundance table of normal, asthma and COPD.

Classification		Normal (n = 12)			Asthma (n = 18)			COPD (n = 17)		
		Totalreads	%	Occurred	Totalreads	%	Occurred	Totalreads	%	Occurred
Phylum	Firmicutes	18067	48.37	12	48172	59.76	17	51317	61.57	17
	Proteobacteria	10840	29.02	12	28702	35.61	17	23376	28.04	16
	Bacteroidetes	6052	16.2	12	2109	2.62	17	5974	7.17	16
	Actinobacteria	2190	5.86	12	1306	1.62	16	1409	1.69	15
	Fusobacteria	167	0.45	1	308	0.38	14	1189	1.43	14
	Cyanobacteria	16	0.45	1	0	0	0	0	0	0
	Spirochaetes	9	0.02	4	2	0	2	2	0	2
	Tenericutes	7	0.02	3	1	0	1	2	0	2
	Acidobacteria	1	0	1	0	0	0	82	0.1	1
	Chloroflexi	0	0	0	4	0	3	3	0	2
Genus	*Streptococcus*	12749	33.05	12	21117	28.34	17	18467	23.52	17
	*Neisseria*	8368	21.69	12	4661	6.26	13	4417	5.62	15
	*Prevotella*	5178	13.42	12	1852	2.49	15	3307	4.21	16
	*Veillonella*	3090	8.01	12	2598	3.49	15	3125	3.98	15
	*Leptotrichia*	2397	6.21	12	151	0.20	14	1076	1.37	14
	*Actinomyces*	1492	3.87	11	551	0.74	14	522	0.66	11
	*Fusobacterium*	696	1.80	10	153	0.21	10	99	0.13	10
	*Porphyromonas*	566	1.47	9	106	0.14	8	106	0.13	8
	*Halomonas*	437	1.13	10	840	1.13	13	359	0.46	12
	*Gemella*	340	0.88	10	249	0.33	13	331	0.42	13
	*Acinetobacter*	109	0.28	7	5	0.01	3	4242	5.40	7
	*Lactobacillus*	26	0.07	6	22109	29.67	14	27093	34.50	15
	*Pseudomonas*	25	0.06	3	11775	15.80	10	7955	10.13	8
	*Enterobacter*	7	0.02	2	983	1.32	9	237	0.30	7
	*Stenotrophomonas*	4	0.01	2	2190	2.94	8	253	0.32	6
	*Citrobacter*	0	0.00	0.00	1319	1.77	3	10.00	0.01	3
	*Leuconostoc*	0	0.00	0.00	901	1.21	4	1090	1.39	6

**Figure 1:**
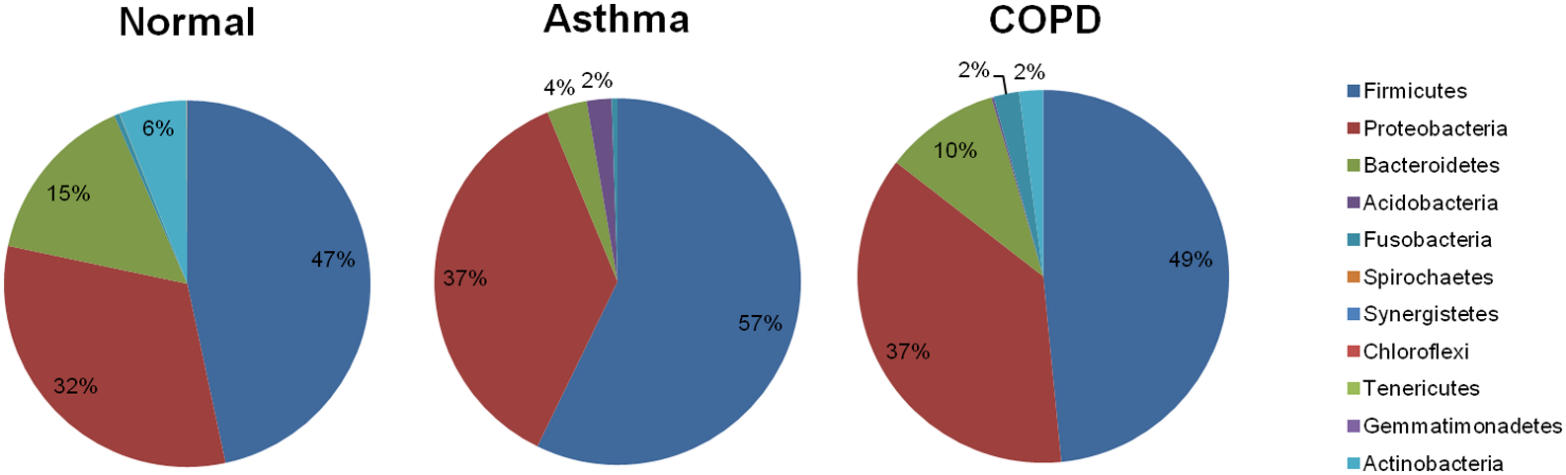
Oropharynx microbial diversity of phyla in the asthma, chronic obstructive pulmonary disease (COPD), and healthy control groups.

The relative abundance of the main phyla identified in the normal control group and in the asthmatic and COPD patients was analyzed using an R script with gplots and the RColorBrewer package (Figure 1). The Firmicutes and Proteobacteria phyla were highly abundant in the asthma and COPD groups. The Bacteroidetes and Actinobacteria phyla had low abundance in the COPD group but were highly abundant in the healthy control group. These results indicate that the increase in microbial communities in the asthma and COPD groups was associated with an abundance of Proteobacteria and Firmicutes along with a reduction in the number of Bacteroidetes.

### Genus identification of bacterial communities

To diversity of the microbial communities present in the oropharyngeal swab samples, was determined from BLASTn analysis, where the assembled sequences showed closest relationship with their neighbouring genera. Although the three data sets showed broad taxonomic similarity, there was notable variation in each sample at the genus level. In total, 190 different bacterial genera were identified in this study: 105, 131, and 133 different genera were present in the healthy control, asthma, and COPD groups, respectively (Table 3). Most sequences in the microbiome library from the healthy control group were *Streptococcus* spp. (33.05%), *Neisseria* spp. (21.69%), *Prevotella* spp. (13.42%), and *Veillonella* spp. (8.01%). Most sequences in the bronchial asthma library were identified as *Lactobacillus* spp. (29.67%), *Streptococcus* spp. (28.34%), *Pseudomonas* spp. (15.80%), *Neisseria* spp. (6.26%), *Prevotella* spp. (2.49%), and *Veillonella* spp. (3.46%). Intriguingly, *Stenotrophomonas* spp. (2.94%) was present in the asthmatic group (eight of 18 patients), but largely absent from the healthy control group (0.01%) and COPD group (0.32%). Most sequences in the COPD library were identified as *Lactobacillus* spp. (34.50%), *Streptococcus* spp. (23.52%), *Pseudomonas* spp. (10.13%), *Neisseria* spp. (5.26%), *Prevotella* spp. (4.21%), and *Veillonella* spp. (3.98%).

In the healthy control group, *Neisseria* spp. (Proteobacteria) and *Prevotella* spp. (Bacteroidetes) constituted 21.69% and 13.42% of the sequences, respectively, and thus comprised more than 40% of the bacterial community in the healthy control group compared to just over 10% in the asthmatic and COPD patients. When compared to the healthy controls, *Lactobacillus* spp. (29.67% and 34.50%) and *Pseudomonas* spp. (15.80% and 10.13%) were much more prevalent in patients with asthma or COPD, respectively. *Lactobacillus* spp. was present in 14 of 18 asthmatic patients and 15 of 17 patients with COPD, and *Pseudomonas* spp. was present in 10 of 18 asthmatic patients and eight of 17 patients with COPD. This was in contrast to the low frequencies of *Lactobacillus* spp. (0.07%) and *Pseudomonas* spp. (0.06%) in the healthy control population, i.e., six and four, respectively, of a total of 12 individuals. The proportion of *Pseudomonas* spp. decreased from 15.80% in the asthma sample to 10.13% in the COPD sample, and that of *Lactobacillus* increased from 29.67% in the asthma sample to 34.50% in the COPD sample. Among the phylum Bacteroidetes, populations of *Prevotella* spp. that were observed in the healthy control group were replaced with *Pseudomonas* spp. of Proteobacteria in the asthma and COPD groups (Table 3). It should be noted that this analysis was coverage-limited.

### Identification by statistical analysis

We found that the three phyla and six genera in the normal controls and patients with asthma and COPD that were identified by sequencing and BLASTn analysis were significantly different in terms of frequency among samples, as indicated by the GLM statistical analysis (Table 4). In the statistical analysis that estimated the number of bacterial genera in each sample, if the value was less than 0, the genus was considered to occur at a greater frequency in the healthy sample. If the value was greater than 0, the genus was considered to occur at a greater frequency in the patient samples. *Pseudomonas* spp. (5.471) and *Lactobacillus* spp. (6.0006) were found with greater frequencies in the asthmatic and COPD patients. *Streptococcus* spp., *Neisseria* spp., *Veillonella* spp., and *Prevotella* spp. were found with greater frequencies in the healthy controls (*p*<0.0001). Jackknife clustering and PCoA were performed using Fast UniFrac [34].

**Table 4:** Statistical analysis of the healthy control, asthma, and chronic obstructive pulmonary disease (COPD) groups.

Phylum	Genus	Estimate		Standard Error		Chi-squared		*p*-value	
		Asthma	COPD	Asthma	COPD	Asthma	COPD	Asthma	COPD
Firmicutes	*Streptococcus* spp.	–0.244	–0.341	0.0112	0.0146	470.19	541.05	<0.0001	<0.0001
Firmicutes	*Lactobacillus* spp.	6.006	6.203	0.1962	0.1965	936.78	996.15	<0.0001	<0.0001
Proteobacteria	*Pseudomonas* spp.	5.471	4.890	0.2002	0.2008	746.37	593.05	<0.0001	<0.0001
Proteobacteria	*Neisseria* spp.	–1.177	–2.069	0.0187	0.0209	3955.36	9841.56	<0.0001	<0.0001
Firmicutes	*Veillonella* spp.	–0.893	–1.191	0.0267	0.0306	1116.00	1517.35	<0.0001	<0.0001
Bacteroidetes	*Prevotella* spp.	–1.773	–1.046	0.0271	0.0303	4284.72	1193.36	<0.0001	<0.0001

**Figure 2:**
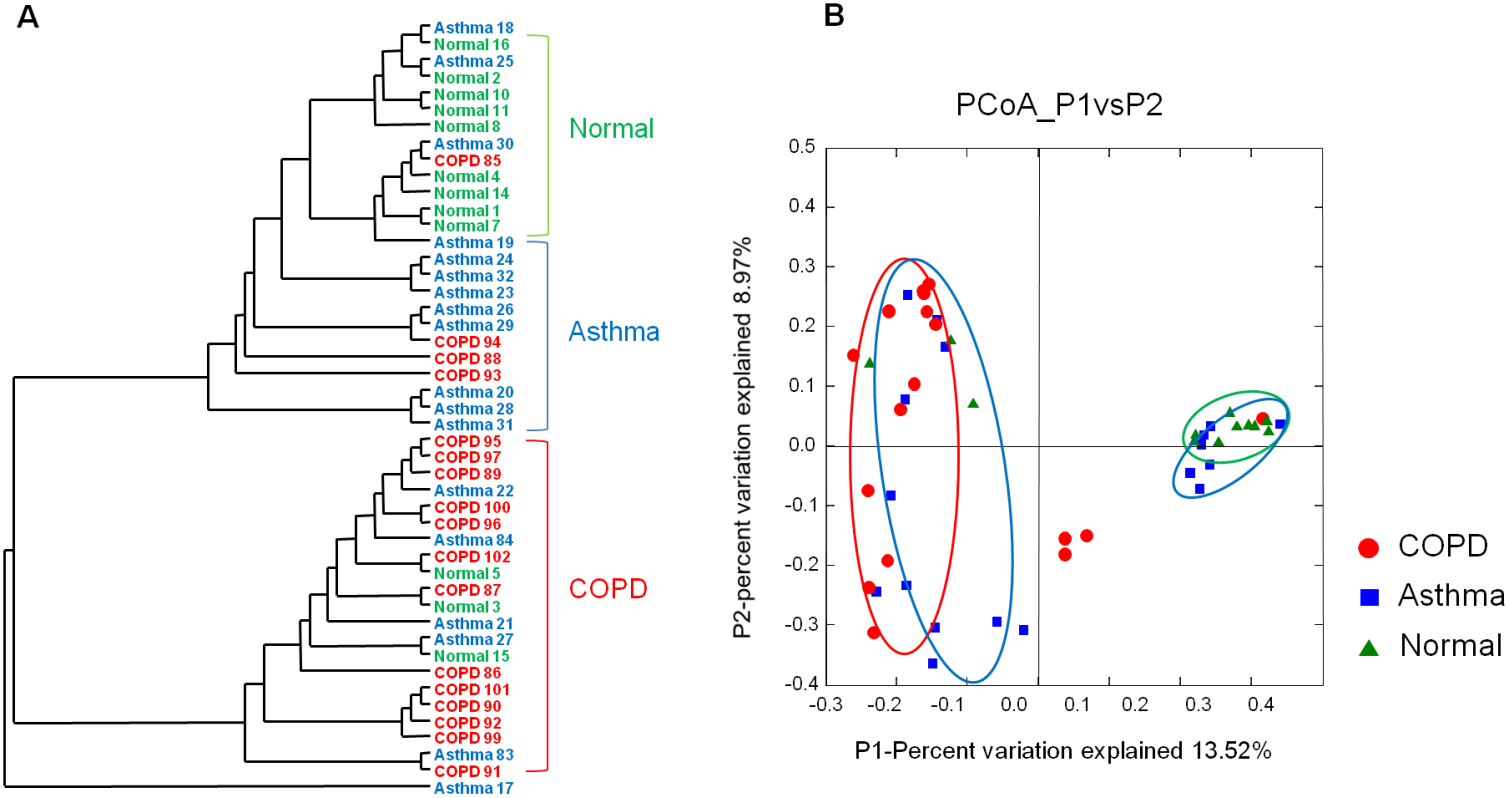
Fast UniFrac analysis of V1–V3 16S sequences of the oropharynx microbial community in the asthma, chronic obstructive pulmonary disease (COPD), and healthy control groups. (A) Jackknife clustering of the environments in the Fast UniFrac dataset. A phylogenetic tree was generated by the neighbor-joining method as implemented in Clustal W with 1,000 bootstrap replicates. The numbers next to the nodes represent the number of times that a particular node was observed in a random sample from the whole dataset. (B) Principal coordinate analysis (PCoA) scatter plot of microbiota from individuals in the healthy control, asthma, and COPD groups (green: healthy control; blue: asthma; red: COPD).

The identification of microbiota in the healthy control, asthma, and COPD groups (Figure 2A), proved to be fairly robust with the Jackknife clustering method (75% bootstrap on all nodes but one). This is also supported by Fast UniFrac PCoA plot results (Figure 2B). The dendrogram and PCoA plots depict well, the differences in overall microbial community structure in each group, highlighting the differences between three groups. It also apparent from these figures that the microbiotas in asthma samples were closer to outlying COPD samples. On the contrary in PCoA plots, asthma samples clustered with both sides of normal and COPD samples. There is a distant overlap between asthma and COPD subjects with many outliers including the controls, emphasizing absence of tight clustering between the disease states. A derangement of some of the samples was observed with asthma no 18, no 25, no 30, and COPD no 85 falling in the normal control cluster, while COPD no 88, no 93, no 94 clustered into the asthma group. Normal control sample no 3, no 5, no 15 and asthma sample no 17, no 21, no 22, no 27, no 83, no 84 were found to be a part of the COPD group.

## Discussion

Respiratory diseases include the common cold, influenza, pharyngitis, sinusitis, bronchitis, pneumonia, asthma, and COPD, which account for more than 10% of visits to primary care physicians [35]. Among these, conditions like asthma and COPD are heterogeneous syndromes of chronic airway inflammation with undetermined pathogenesis and high mortality rates [36]. There are many guidelines for the diagnosis and management of asthma and COPD, but to date both diseases have often been misdiagnosed and undertreated [36], [37]. The key to confront this problem is to understand and characterize the microbiome of these disease conditions in comparison to the healthy state. The human microbiome studies can identify the link between healthy and diseased states and have the capacity to reveal disease mechanisms.

So far the studies of the microbial community in asthma and COPD of human airways, have targeted the lower respiratory tracts using samples such as BAL and the sputum, showing controversial results about the role of the microbiota. There are still gaps when compared to previous studies in this aspect, for instance trying to understand the role of the microbial populations in the lower respiratory tract of oropharyngeal airway in conditions like asthma and COPD which is still unclear. So it becomes imperative to get a true representation of the microbiome in these disease states.

In present decade it has become possible to capture the true microbial diversity with modern high throughput culture-independent techniques which aid in comparing the microbiome between the healthy and diseased conditions. These studies can provide new information on the oropharyngeal microbiota in asthma and COPD patients. Previously, several studies investigated the links between airway bacterial concentrations and COPD, but with targeted molecular typing methods aimed at detecting specific bacterial species in the sputum [10], [11]. These studies demonstrated that changes in the bacterial load in the sputum were unlikely to be an important mechanism in the exacerbation of COPD. These results were also supported by a study of the microbiome of healthy smokers and individuals with COPD using 454 pyrosequencing analysis, which showed that relatively low numbers of diverse types of bacteria existed in the lung [19].

In the current study, we gathered comprehensive information concentrating on the whole microbial diversity of the lower respiratory tract as opposed to previous studies with a non-targeted culture independent approach. We have three main conclusions from this study. First in terms of overall diversity, the oropharyngeal airway microbiota are quite diverse but reveal only few differences between asthma and COPD patients. Secondly, *Pseudomonas* spp. of the phylum Proteobacteria and *Lactobacillus* spp. of the phylum Firmicutes prove to be the most dominant populations in asthma and COPD patients but however were not detected in the healthy oropharynx. The final conclusion from this study is the dominance of groups like, *Streptococcus*, *Veillonella*, *Prevotella*, and *Neisseria*, belonging to the phylum Bacteroidetes, in the healthy oropharynx.

Briefly highlighting the methodology, sequences were generated using a high-throughput GS-FLX 454 pyrosequencer and aligned using tools like PRINSEQ, RDP, and Clustal_X. A GLM analysis of the 259,572 total sequence reads reveals a rich microbial environment in the oropharyngeal airway in asthma and COPD individuals. Distance-based statistical UniFrac tests were used for detecting the changes in abundance and rare lineages [33]. Minimal differences were observed between asthma and COPD individuals (Proteobacteria, Firmicutes; *p*<0.0001). As observed *Pseudomonas* (estimated asthma: 5.471; COPD: 4.890) and *Lactobacillus* spp. (estimated asthma: 6.006; COPD: 6.203) were found to be in abundance in asthma and COPD subjects when compared to healthy individuals. This is consistent with the findings of previous diversity studies in the bronchi of asthma and COPD patients, by 16S rRNA gene-based Sanger sequencing and by the 16S rRNA PhyloChip methods in endotracheal aspirates [38], [39].

Hilty et al. [39] analyzed 5,054 16S rRNA bacterial clones in the bronchi and found that the members of Proteobacteria were in dominance in asthmatic children, whereas Bacteroidetes, particularly *Prevotella*, were more frequent in controls than in adult or pediatric asthmatic or COPD patients. Sze et al. [40] evaluated the lung tissue microbiomes of patients with severe COPD at the time of lung transplantation and noted increased bacterial diversity of the phylum Firmicutes, attributable to an increase in *Lactobacillus* species. Using 16S rRNA-based culture-independent profiling methods, Huang *et al.* [41] reported increased microbiota at exacerbation of COPD primarily belonging to phylum Proteobacteria, including some non-typical COPD pathogens. In another study a combination of high-density 16S ribosomal RNA microarray and parallel clone library-sequencing analysis in asthmatic patients revealed a rich microbial environment in the oropharyngeal airway [42].

In the oropharyngeal airway of asthma and COPD patients, *Pseudomonas*, *Stenotrophomonas*, and *Lactobacillus* were found to be dominant. Whereas the main phyla reported in the bronchoalvelolar lavage fluid of COPD patients are Actinobacteria, Firmicutes, and Proteobacteria, with dominance of *Pseudomonas*, *Haemophilus*, or *Stenotrophomonas*. in the respective phyla [19]. However, *Haemophilus* was not found in our study of the oropharynx. *Pseudomonas* and *Stenotrophomonas maltophilia* are the most common pathogenic bacteria associated with cystic fibrosis [43]–[45]. In COPD, *Pseudomonas* was also reported to a risk factor for severe acute exacerbation [19] and *Lactobacillus* is known to be involved in severe COPD and fatal lung abscess [19], [40]. Therefore, further study is necessary to understand the pathogenesis of diseases at microanatomic sites of the respiratory tract.

In our study the healthy controls showed higher frequencies of organisms from *Streptococcus*, *Veillonella*, *Prevotella*, and *Neisseria* of phylum Firmicutes when compared to asthmatic and COPD patients. Normally, *Streptococcus* species are considered to be commensal bacteria of the oral cavity; however, *S. pneumoniae*, *S. mitis*, and *S. oralis* are members of the viridians streptococci and are associated with pneumonia and endocarditis and are considered as opportunistic pathogens [46]–[49]. *Veillonella* is a type of subgingival microflora found at periodontal disease sites and is known to be a periodontopathogenic bacteria [50]. Recently, *Prevotella copri* was reported to be involved in inflammation [51].

Previously, several studies reported less abundance of *Neisseria* in smokers [16], [52], [53]. This study also showed that *Neisseria* was less dominant in asthmatic and COPD patients, implying that *Neisseria* is an important indicator of the health of the oral and upper respiratory tract. Therefore, the potential relationship between these oropharynx microbiota and chronic respiratory diseases should be evaluated.

Previously exquisite study of the bronchial microbiome obtained from lower respiratory samples avoiding upper airway contamination in the healthy adults revealed overlapping of certain groups of bacteria similar to upper airways in six subjects [16]. Therefore, our results suggest that the distribution and profile of microbiota are consistent between the oropharynx and bronchi in asthma and COPD patients and that the pathogenic role of oropharyngeal commensal bacteria changes with n the progression of chronic respiratory diseases.

## Supporting Information

Figure S1 **Bacterial diversity in respiratory tract samples.** The graph shows rarefaction curves indicating the number of assigned operational taxonomic units in relation to the number of 16S rRNA sequences.
(TIF)

Table S1 **Abundance table of normal, asthma and COPD.**
(DOC)

## References

[1] Janeiro RD (2013) Differentiating chronic obstructive pulmonary disease from asthma in clinical practice. HUPE Journal 12: 57–64.

[2] World Health Organization, Office of Health Communications and Public Relations. (2006) Asthma. Geneva: World Health Organization. 3 p.

[3] Mathers CD, Loncar D (2006) Projections of global mortality and burden of disease from 2002 to 2030. PLoS Med 3: e442. 17132052 10.1371/journal.pmed.0030442 PMC1664601

[4] World Health Organization “The 10 leading causes of death in the world, 2000 and 2011”, July 2013. Retrieved 2013 November 29.

[5] Kurai D, Saraya T, Ishii H, Takizawa H (2013) Virus-induced exacerbations in asthma and COPD. Front Microbiol 4: 293. 24098299 10.3389/fmicb.2013.00293 PMC3787546

[6] Johnston SL, Martin RJ (2005) *Chlamydophila pneumoniae* and *Mycoplasma pneumoniae*: a role in asthma pathogenesis? Am J Respir Crit Care Med 172: 1078–1089. 15961690 10.1164/rccm.200412-1743PP

[7] Kraft M, Cassell GH, Pak J, Martin RJ (2002) *Mycoplasma pneumoniae* and *Chlamydia pneumoniae* in asthma: effect of clarithromycin. Chest 121: 1782–1788. 12065339 10.1378/chest.121.6.1782

[8] Kraft M, Adler KB, Ingram JL, Crews AL, Atkinson TP, et al (2008) *Mycoplasma pneumoniae* induces airway epithelial cell expression of MUC5AC in asthma. Eur Respir J 31: 43–46. 18166592 10.1183/09031936.00103307

[9] Alamoudi OS (2007) Bacterial infection and risk factors in outpatients with acute exacerbation of chronic obstructive pulmonary disease: a 2-year prospective study. Respirology 12: 283–287. 17298464 10.1111/j.1440-1843.2006.01002.x

[10] Dang HT, Kim SA, Park HK, Shin JW, Park SG, et al (2013) Analysis of Oropharyngeal Microbiota between the Patients with Bronchial Asthma and the Non-Asthmatic Persons. J Bacteriol Virol 43: 270–278.

[11] Sethi S, Sethi R, Eschberger K, Lobbins P, Cai X, et al (2007) Airway bacterial concentrations and exacerbations of chronic obstructive pulmonary disease. Am J Respir Crit Care Med 176: 356–361. 17478618 10.1164/rccm.200703-417OC

[12] Bourdin A, Gras D, Vachier I, Chanez P (2009) Upper airway x 1: allergic rhinitis and asthma: united disease through epithelial cells. Thorax 64: 999–1004. 19864543 10.1136/thx.2008.112862

[13] Lemon KP, Klepac-Ceraj V, Schiffer HK, Brodie EL, Lynch SV, et al. (2010) Comparative analyses of the bacterial microbiota of the human nostril and oropharynx. MBio 1. 10.1128/mBio.00129-10 PMC2925076 20802827

[14] Gerritsen J, Smidt H, Rijkers GT, de Vos WM (2011) Intestinal microbiota in human health and disease: the impact of probiotics. Genes Nutr 6: 209–240. 21617937 10.1007/s12263-011-0229-7 PMC3145058

[15] Ramsey CD, Gold DR, Litonjua AA, Sredl DL, Ryan L, et al (2007) Respiratory illnesses in early life and asthma and atopy in childhood. J Allergy Clin Immunol 119: 150–156. 17208596 10.1016/j.jaci.2006.09.012

[16] Charlson ES, Bittinger K, Haas AR, Fitzgerald AS, Frank I, et al (2011) Topographical continuity of bacterial populations in the healthy human respiratory tract. Am J Respir Crit Care Med 184: 957–963. 21680950 10.1164/rccm.201104-0655OC PMC3208663

[17] Ramsey CD, Gold DR, Litonjua AA, Sredl DL, Ryan L, et al (2007) Respiratory illnesses in early life and asthma and atopy in childhood. J Allergy Clin Immunol 119: 150–156. 17208596 10.1016/j.jaci.2006.09.012

[18] Erb-Downward JR, Thompson DL, Han MK, Freeman CM, McCloskey L, et al (2011) Analysis of the lung microbiome in the “healthy” smoker and in COPD. PLoS One 6: e16384. 21364979 10.1371/journal.pone.0016384 PMC3043049

[19] Garzoni C, Brugger SD, Qi W, Wasmer S, Cusini A, et al (2013) Microbial communities in the respiratory tract of patients with interstitial lung disease. Thorax 68: 1150–1156. 23945167 10.1136/thoraxjnl-2012-202917 PMC3841796

[20] Noverr MC, Huffnagle GB (2005) The ‘microflora hypothesis’ of allergic diseases. Clin Exp Allergy 35: 1511–1520. 16393316 10.1111/j.1365-2222.2005.02379.x

[21] Jumpstart Consortium Human Microbiome Project Data Generation Working Group (2012) Evaluation of 16S rDNA-based community profiling for human microbiome research. PLoS One 7: e39315. 22720093 10.1371/journal.pone.0039315 PMC3374619

[22] Li K, Bihan M, Yooseph S, Methe BA (2012) Analyses of the microbial diversity across the human microbiome. PLoS One 7: e32118. 22719823 10.1371/journal.pone.0032118 PMC3374608

[23] British Thoracic Society (2003) British guideline on the management of asthma. Scottish Intercollegiate Guidelines Network. Thorax 58: 1–94.

[24] National Institutes of Health (1997) Guidelines for the diagnosis and management of asthma. National Asthma Education and Prevention Program Expert Panel Report 2. Z Naturforsch C. Washington, DC: U.S. Government Printing Office. 97–4051.

[25] Pauwels RA, Buist AS, Calverley PM, Jenkins CR, Hurd SS (2001) Global strategy for the diagnosis, management, and prevention of chronic obstructive pulmonary disease. NHLBI/WHO Global Initiative for Chronic Obstructive Lung Disease (GOLD) Workshop summary. Am J Respir Crit Care Med 163: 1256–1276. 11316667 10.1164/ajrccm.163.5.2101039

[26] Ausubel FM, Brent R, Kingston RE, Moore DD, Seidman JGS, et al. (1993) Miniprep of bacterial genomic DNA. Current Protocols in Molecular Biology. New York. 2.4. p.

[27] Woese CR (1987) Bacterial evolution. Microbiol Rev 51: 221–271. 2439888 10.1128/mr.51.2.221-271.1987 PMC373105

[28] Schmieder R, Edwards R (2011) Quality control and preprocessing of metagenomic datasets. Bioinformatics 27: 863–864. 21278185 10.1093/bioinformatics/btr026 PMC3051327

[29] Cole JR, Chai B, Farris RJ, Wang Q, Kulam-Syed-Mohideen AS, et al (2007) The ribosomal database project (RDP-II): introducing myRDP space and quality controlled public data. Nucleic Acids Res 35: D169–172. 17090583 10.1093/nar/gkl889 PMC1669760

[30] Thompson JD, Gibson TJ, Plewniak F, Jeanmougin F, Higgins DG (1997) The CLUSTAL_X windows interface: flexible strategies for multiple sequence alignment aided by quality analysis tools. Nucleic Acids Res 25: 4876–4882. 9396791 10.1093/nar/25.24.4876 PMC147148

[31] Lozupone C, Knight R (2005) UniFrac: a new phylogenetic method for comparing microbial communities. Appl Environ Microbiol 71: 8228–8235. 16332807 10.1128/AEM.71.12.8228-8235.2005 PMC1317376

[32] Lozupone C, Hamady M, Knight R (2006) UniFrac–an online tool for comparing microbial community diversity in a phylogenetic context. BMC Bioinformatics 7: 371. 16893466 10.1186/1471-2105-7-371 PMC1564154

[33] Chen J, Bittinger K, Charlson ES, Hoffmann C, Lewis J, et al (2012) Associating microbiome composition with environmental covariates using generalized UniFrac distances. Bioinformatics 28: 2106–2113. 22711789 10.1093/bioinformatics/bts342 PMC3413390

[34] Hamady M, Lozupone C, Knight R (2010) Fast UniFrac: facilitating high-throughput phylogenetic analyses of microbial communities including analysis of pyrosequencing and PhyloChip data. ISME J 4: 17–27. 19710709 10.1038/ismej.2009.97 PMC2797552

[35] Bates JH, Campbell GD, Barron AL, McCracken GA, Morgan PN, et al (1992) Microbial etiology of acute pneumonia in hospitalized patients. Chest 101: 1005–1012. 1555415 10.1378/chest.101.4.1005

[36] Buist AS (2003) Similarities and differences between asthma and chronic obstructive pulmonary disease: treatment and early outcomes. Eur Respir J Suppl 39: 30s–35s. 10.1183/09031936.03.00404903 12572699

[37] Athanazio R (2012) Airway disease: similarities and differences between asthma, COPD and bronchiectasis. Clinics (Sao Paulo) 67: 1335–1343. 23184213 10.6061/clinics/2012(11)19 PMC3488995

[38] Huang YJ, Kim E, Cox MJ, Brodie EL, Brown R, et al (2010) A persistent and diverse airway microbiota present during chronic obstructive pulmonary disease exacerbations. OMICS 14: 9–59. 20141328 10.1089/omi.2009.0100 PMC3116451

[39] Hilty M, Burke C, Pedro H, Cardenas P, Bush A, et al (2010) Disordered microbial communities in asthmatic airways. PLoS One 5: e8578. 20052417 10.1371/journal.pone.0008578 PMC2798952

[40] Sze MA, Dimitriu PA, Hayashi S, Elliott WM, McDonough JE, et al (2012) The lung tissue microbiome in chronic obstructive pulmonary disease. Am J Respir Crit Care Med 185: 1073–1080. 22427533 10.1164/rccm.201111-2075OC PMC3359894

[41] Huang YJ, Sethi S, Murphy T, Nariya S, Boushey HA, et al (2014) Airway Microbiome Dynamics in Exacerbations of Chronic Obstructive Pulmonary Disease. J Clin Microbiol. 10.1128/JCM.00035-14 PMC4136157 24850358

[42] Huang YJ, Nelson CE, Brodie EL, Desantis TZ, Baek MS, et al (2011) Airway microbiota and bronchial hyperresponsiveness in patients with suboptimally controlled asthma. J Allergy Clin Immunol 127: 372–381 e371–373. 21194740 10.1016/j.jaci.2010.10.048 PMC3037020

[43] de Vrankrijker AM, Wolfs TF, van der Ent CK (2010) Challenging and emerging pathogens in cystic fibrosis. Paediatr Respir Rev 11: 246–254. 21109184 10.1016/j.prrv.2010.07.003

[44] Emerson J, McNamara S, Buccat AM, Worrell K, Burns JL (2010) Changes in cystic fibrosis sputum microbiology in the United States between 1995 and 2008. Pediatr Pulmonol 45: 363–370. 20232473 10.1002/ppul.21198

[45] Millar FA, Simmonds NJ, Hodson ME (2009) Trends in pathogens colonising the respiratory tract of adult patients with cystic fibrosis, 1985–2005. J Cyst Fibros 8: 386–391. 19740710 10.1016/j.jcf.2009.08.003

[46] Balkundi DR, Murray DL, Patterson MJ, Gera R, Scott-Emuakpor A, et al (1997) Penicillin-resistant *Streptococcus mitis* as a cause of septicemia with meningitis in febrile neutropenic children. J Pediatr Hematol Oncol 19: 82–85. 9065725 10.1097/00043426-199701000-00013

[47] Cabellos C, Viladrich PF, Corredoira J, Verdaguer R, Ariza J, et al (1999) *Streptococcal meningitis* in adult patients: current epidemiology and clinical spectrum. Clin Infect Dis 28: 1104–1108. 10452643 10.1086/514758

[48] Lu HZ, Weng XH, Zhu B, Li H, Yin YK, et al (2003) Major outbreak of toxic shock-like syndrome caused by *Streptococcus mitis* . J Clin Microbiol 41: 3051–3055. 12843042 10.1128/JCM.41.7.3051-3055.2003 PMC165286

[49] Lyytikainen O, Rautio M, Carlson P, Anttila VJ, Vuento R, et al (2004) Nosocomial bloodstream infections due to viridans streptococci in haematological and non-haematological patients: species distribution and antimicrobial resistance. J Antimicrob Chemother 53: 631–634. 15014065 10.1093/jac/dkh159

[50] Socransky SS, Haffajee AD, Cugini MA, Smith C, Kent RL Jr (1998) Microbial complexes in subgingival plaque. J Clin Periodontol 25: 134–144. 9495612 10.1111/j.1600-051x.1998.tb02419.x

[51] Hofer U (2014) Microbiome: pro-inflammatory Prevotella? Nat Rev Microbiol 12: 5. 24270843 10.1038/nrmicro3180

[52] Tamada K, Tomonaga S, Hatanaka F, Nakai N, Takao K, et al (2010) Decreased exploratory activity in a mouse model of 15q duplication syndrome; implications for disturbance of serotonin signaling. PLoS One 5: e15126. 21179543 10.1371/journal.pone.0015126 PMC3002297

[53] Morris A, Beck JM, Schloss PD, Campbell TB, Crothers K, et al (2013) Comparison of the respiratory microbiome in healthy nonsmokers and smokers. Am J Respir Crit Care Med 187: 1067–1075. 23491408 10.1164/rccm.201210-1913OC PMC3734620

