# Supplementary figures and images for: Microbial Communities in the Upper Respiratory Tract of Patients with Asthma and Chronic Obstructive Pulmonary Disease
Source: PLoS One. 2014 Oct 16;9(10):e109710. doi: 10.1371/journal.pone.0109710 (PMC4199592; doi:10.1371/journal.pone.0109710)

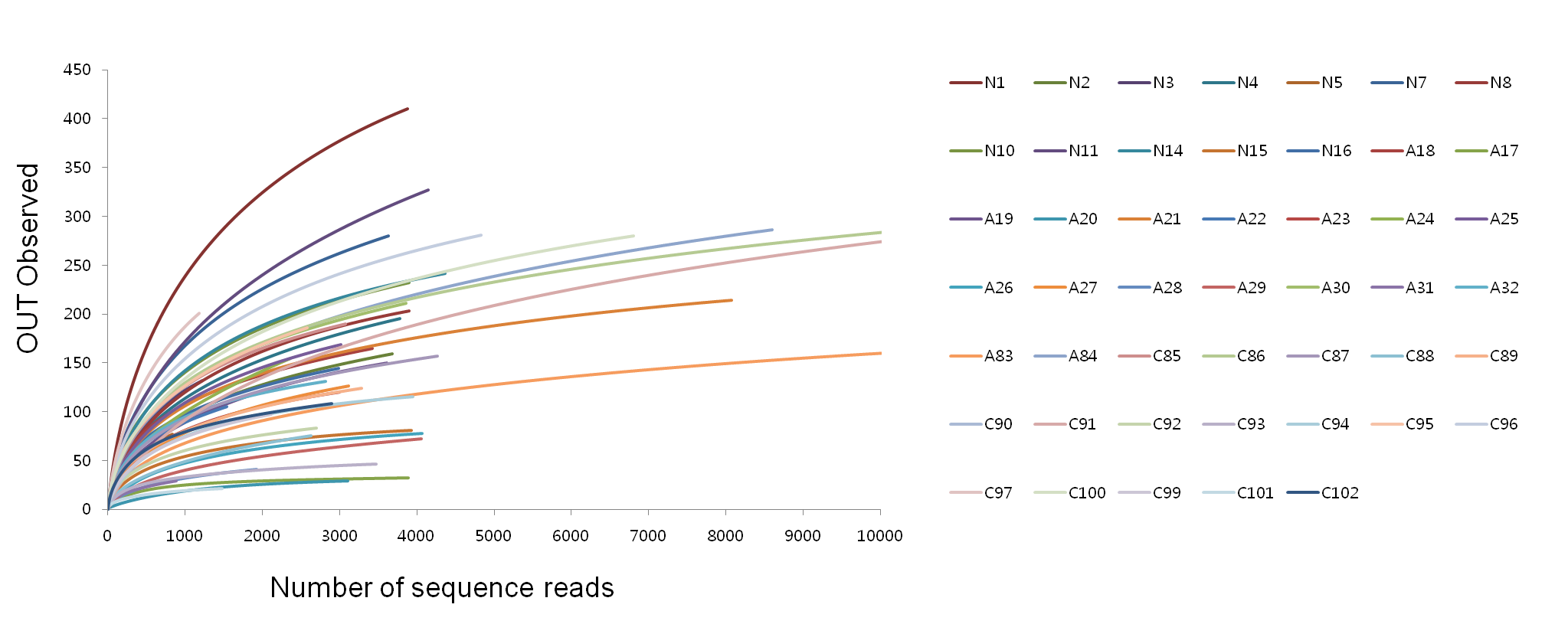

Supplement: Figure S1 — Bacterial diversity in respiratory tract samples. The graph shows rarefaction curves indicating the number of assigned operational taxonomic units in relation to the number of 16S rRNA sequences. (TIF) [file pone.0109710.s001.tif]
