# Supplementary material for: Microbial Communities in the Upper Respiratory Tract of Patients with Asthma and Chronic Obstructive Pulmonary Disease
Source: PLoS One. 2014 Oct 16;9(10):e109710. doi: 10.1371/journal.pone.0109710 (PMC4199592; doi:10.1371/journal.pone.0109710)
Supplement: Table S1 — Abundance table of normal, asthma and COPD. (DOC) [file pone.0109710.s002.doc]

**Table S1. Abundance table of normal, asthma and COPD**

| Classification | | Normal (12) | | | Asthma (18) | | | COPD (17) | | |
| --- | --- | --- | --- | --- | --- | --- | --- | --- | --- | --- |
|  |  | Total reads | % | Occurred | Total reads | % | Occurred | Total reads | % | Occurred |
| Phylum | Firmicutes | 18067 | 48.37 | 12 | 48172 | 59.76 | 17 | 51317 | 61.57 | 17 |
|  | Proteobacteria | 10840 | 29.02 | 12 | 28702 | 35.61 | 17 | 23376 | 28.04 | 16 |
|  | Bacteroidetes | 6052 | 16.2 | 12 | 2109 | 2.62 | 17 | 5974 | 7.17 | 16 |
|  | Actinobacteria | 2190 | 5.86 | 12 | 1306 | 1.62 | 16 | 1409 | 1.69 | 15 |
|  | Fusobacteria | 167 | 0.45 | 1 | 308 | 0.38 | 14 | 1189 | 1.43 | 14 |
|  | Cyanobacteria | 16 | 0.45 | 1 | 0 | 0 | 0 | 0 | 0 | 0 |
|  | Spirochaetes | 9 | 0.02 | 4 | 2 | 0 | 2 | 2 | 0 | 2 |
|  | Tenericutes | 7 | 0.02 | 3 | 1 | 0 | 1 | 2 | 0 | 2 |
|  | Acidobacteria | 1 | 0 | 1 | 0 | 0 | 0 | 82 | 0.1 | 1 |
|  | Chloroflexi | 0 | 0 | 0 | 4 | 0 | 3 | 3 | 0 | 2 |
|  | Gemmatimonadetes | 0 | 0 | 0 | 1 | 0 | 1 | 0 | 0 | 0 |
|  | Synergistetes | 0 | 0 | 0 | 2 | 0 | 1 | 0 | 0 | 0 |
| Genus | *Streptococcus* | 12749 | 33.05 | 12(12) | 21117 | 28.34 | 17(18) | 18467 | 23.52 | 17(17) |
|  | *Neisseria* | 8368 | 21.69 | 12(12) | 4661 | 6.26 | 13(18) | 4417 | 5.62 | 15(17) |
|  | *Prevotella* | 5178 | 13.42 | 12(12) | 1852 | 2.49 | 15(18) | 3307 | 4.21 | 16(17) |
|  | *Veillonella* | 3090 | 8.01 | 12(12) | 2598 | 3.49 | 15(18) | 3125 | 3.98 | 15(17) |
|  | *Leptotrichia* | 2397 | 6.21 | 12(12) | 151 | 0.20 | 14(18) | 1076 | 1.37 | 14(17) |
|  | *Actinomyces* | 1492 | 3.87 | 11(12) | 551 | 0.74 | 14(18) | 522 | 0.66 | 11(17) |
|  | *Fusobacterium* | 696 | 1.80 | 10(12) | 153 | 0.21 | 10(18) | 99 | 0.13 | 10(17) |
|  | *Porphyromonas* | 566 | 1.47 | 9(12) | 106 | 0.14 | 8(18) | 106 | 0.13 | 8(17) |
|  | *Halomonas* | 437 | 1.13 | 10(12) | 840 | 1.13 | 13(18) | 359 | 0.46 | 12(17) |
|  | *Gemella* | 340 | 0.88 | 10(12) | 249 | 0.33 | 13(18) | 331 | 0.42 | 13(17) |
|  | *Megasphaera* | 328 | 0.85 | 8(12) | 33 | 0.04 | 6(18) | 31 | 0.04 | 8(17) |
|  | *Haemophilus* | 306 | 0.79 | 11(12) | 320 | 0.43 | 11(18) | 77 | 0.10 | 10(17) |
|  | *Rothia* | 284 | 0.74 | 12(12) | 434 | 0.58 | 14(18) | 724 | 0.92 | 14(17) |
|  | *Johnsonella* | 279 | 0.72 | 10(12) | 155 | 0.21 | 9(18) | 117 | 0.15 | 9(17) |
|  | *Granulicatella* | 227 | 0.59 | 11(12) | 194 | 0.26 | 11(18) | 81 | 0.10 | 11(17) |
|  | *Capnocytophaga* | 225 | 0.58 | 11(12) | 121 | 0.16 | 10(18) | 59 | 0.08 | 10(17) |
|  | *Moryella* | 205 | 0.53 | 10(12) | 76 | 0.10 | 11(18) | 30 | 0.04 | 8(17) |
|  | *Atopobium* | 204 | 0.53 | 8(12) | 244 | 0.33 | 9(18) | 70 | 0.09 | 9(17) |
|  | *Oribacterium* | 174 | 0.45 | 11(12) | 104 | 0.14 | 11(18) | 137 | 0.17 | 11(17) |
|  | *Campylobacter* | 144 | 0.37 | 10(12) | 69 | 0.09 | 10(18) | 46 | 0.06 | 8(17) |
|  | *Acinetobacter* | 109 | 0.28 | 7(12) | 5 | 0.01 | 3(18) | 4242 | 5.40 | 7(17) |
|  | *Subdoligranulum* | 78 | 0.20 | 7(12) | 36 | 0.05 | 9(18) | 14 | 0.02 | 5(17) |
|  | *Corynebacterium* | 55 | 0.14 | 10(12) | 29 | 0.04 | 5(18) | 24 | 0.03 | 9(17) |
|  | *Derxia* | 52 | 0.13 | 4(12) | 10 | 0.01 | 3(18) | 11 | 0.01 | 2(17) |
|  | *Solobacterium* | 51 | 0.13 | 9(12) | 20 | 0.03 | 4(18) | 19 | 0.02 | 6(17) |
|  | *Selenomonas* | 40 | 0.10 | 9(12) | 16 | 0.02 | 7(18) | 14 | 0.02 | 7(17) |
|  | *Actinobacillus* | 38 | 0.10 | 2(12) | 1 | 0.00 | 1(18) | 631 | 0.80 | 8(17) |
|  | *Mogibacterium* | 32 | 0.08 | 6(12) | 17 | 0.02 | 6(18) | 7 | 0.01 | 5(17) |
|  | *Lactobacillus* | 26 | 0.07 | 6(12) | 22109 | 29.67 | 14(18) | 27093 | 34.50 | 15(17) |
|  | *Pseudomonas* | 25 | 0.06 | 3(12) | 11775 | 15.80 | 10(18) | 7955 | 10.13 | 8(17) |
|  | *Catonella* | 25 | 0.06 | 4(12) | 13 | 0.02 | 7(18) | 8 | 0.01 | 3(17) |
|  | *Blautia* | 24 | 0.06 | 8(12) | 95 | 0.13 | 11(18) | 184 | 0.23 | 11(17) |
|  | *Bacteroides* | 17 | 0.04 | 7(12) | 11 | 0.01 | 4(18) | 30 | 0.04 | 8(17) |
|  | *Ornithinimicrobium* | 16 | 0.04 | 8(12) | 11 | 0.01 | 4(18) | 4 | 0.01 | 3(17) |
|  | *Filifactor* | 16 | 0.04 | 3(12) | 5 | 0.01 | 3(18) | 0 | 0.00 | 0.00 |
|  | *Comamonas* | 15 | 0.04 | 5(12) | 0 | 0.00 | 0.00 | 3 | 0.00 | 3(17) |
|  | *Vibrio* | 13 | 0.03 | 3(12) | 21 | 0.03 | 5(18) | 13 | 0.02 | 7(17) |
|  | *Dialister* | 13 | 0.03 | 6(12) | 5 | 0.01 | 4(18) | 21 | 0.03 | 7(17) |
|  | *Dermatophilus* | 11 | 0.03 | 4(12) | 10 | 0.01 | 2(18) | 9 | 0.01 | 3(17) |
|  | *Bergeyella* | 11 | 0.03 | 4(12) | 8 | 0.01 | 2(18) | 2 | 0.00 | 2(17) |
|  | *Actinobaculum* | 11 | 0.03 | 2(12) | 1 | 0.00 | 1(18) | 0 | 0.00 | 0.00 |
|  | *Xylanibacter* | 10 | 0.03 | 3(12) | 1 | 0.00 | 1(18) | 3 | 0.00 | 2(17) |
|  | *Kingella* | 10 | 0.03 | 2(12) | 0 | 0.00 | 0.00 | 10 | 0.01 | 1(17) |
|  | *Abiotrophia* | 9 | 0.02 | 3(12) | 7 | 0.01 | 2(18) | 73 | 0.09 | 7(17) |
|  | *Frondihabitans* | 9 | 0.02 | 6(12) | 7 | 0.01 | 4(18) | 8 | 0.01 | 7(17) |
|  | *Butyrivibrio* | 9 | 0.02 | 3(12) | 4 | 0.01 | 2(18) | 13 | 0.02 | 3(17) |
|  | *Treponema* | 9 | 0.02 | 4(12) | 2 | 0.00 | 2(18) | 2 | 0.00 | 2(17) |
|  | *Tannerella* | 9 | 0.02 | 3(12) | 2 | 0.00 | 1(18) | 0 | 0.00 | 0.00 |
|  | *Eikenella* | 8 | 0.02 | 2(12) | 0 | 0.00 | 0.00 | 0 | 0.00 | 0.00 |
|  | *Enterobacter* | 7 | 0.02 | 2(12) | 983 | 1.32 | 9(18) | 237 | 0.30 | 7(17) |
|  | *Paenibacillus* | 7 | 0.02 | 4(12) | 3 | 0.00 | 3(18) | 4 | 0.01 | 3(17) |
|  | *Ralstonia* | 7 | 0.02 | 2(12) | 2 | 0.00 | 1(18) | 0 | 0.00 | 0.00 |
|  | *Marinobacter* | 6 | 0.02 | 2(12) | 7 | 0.01 | 3(18) | 4 | 0.01 | 2(17) |
|  | *Conchiformibius* | 6 | 0.02 | 3(12) | 6 | 0.01 | 4(18) | 6 | 0.01 | 3(17) |
|  | *Paludibacter* | 6 | 0.02 | 3(12) | 1 | 0.00 | 1(18) | 2 | 0.00 | 2(17) |
|  | *Leucobacter* | 5 | 0.01 | 4(12) | 2 | 0.00 | 2(18) | 5 | 0.01 | 3(17) |
|  | *Propionibacterium* | 5 | 0.01 | 3(12) | 2 | 0.00 | 2(18) | 5 | 0.01 | 2(17) |
|  | *Thermobacillus* | 5 | 0.01 | 4(12) | 2 | 0.00 | 1(18) | 5 | 0.01 | 3(17) |
|  | *Peptostreptococcus* | 5 | 0.01 | 2(12) | 1 | 0.00 | 1(18) | 1 | 0.00 | 1(17) |
|  | *Stenotrophomonas* | 4 | 0.01 | 2(12) | 2190 | 2.94 | 8(18) | 253 | 0.32 | 6(17) |
|  | *Providencia* | 4 | 0.01 | 3(12) | 8 | 0.01 | 3(18) | 3 | 0.00 | 3(17) |
|  | *Mycoplasma* | 4 | 0.01 | 2(12) | 1 | 0.00 | 1(18) | 1 | 0.00 | 1(17) |
|  | *Staphylococcus* | 3 | 0.01 | 3(12) | 21 | 0.03 | 9(18) | 133 | 0.17 | 6(17) |
|  | *Faecalibacterium* | 3 | 0.01 | 2(12) | 5 | 0.01 | 2(18) | 1 | 0.00 | 1(17) |
|  | *Saccharomonospora* | 3 | 0.01 | 3(12) | 3 | 0.00 | 3(18) | 1 | 0.00 | 1(17) |
|  | *Aggregatibacter* | 3 | 0.01 | 2(12) | 1 | 0.00 | 1(18) | 20 | 0.03 | 6(17) |
|  | *Brevibacterium* | 3 | 0.01 | 3(12) | 0 | 0.00 | 0.00 | 3 | 0.00 | 2(17) |
|  | *Zoogloea* | 3 | 0.01 | 3(12) | 0 | 0.00 | 0.00 | 0 | 0.00 | 0.00 |
|  | *Lactococcus* | 2 | 0.01 | 1(12) | 6 | 0.01 | 3(18) | 12 | 0.02 | 3(17) |
|  | *Variovorax* | 2 | 0.01 | 2(12) | 5 | 0.01 | 3(18) | 17 | 0.02 | 3(17) |
|  | *Streptomyces* | 2 | 0.01 | 2(12) | 3 | 0.00 | 2(18) | 21 | 0.03 | 6(17) |
|  | *Sutterella* | 2 | 0.01 | 1(12) | 1 | 0.00 | 1(18) | 0 | 0.00 | 0.00 |
|  | *Alcaligenes* | 2 | 0.01 | 2(12) | 0 | 0.00 | 0.00 | 0 | 0.00 | 0.00 |
|  | *Brevundimonas* | 2 | 0.01 | 1(12) | 0 | 0.00 | 0.00 | 0 | 0.00 | 0.00 |
|  | *Bulleidia* | 2 | 0.01 | 1(12) | 0 | 0.00 | 0.00 | 0 | 0.00 | 0.00 |
|  | *Bacillus* | 1 | 0.00 | 1(12) | 59 | 0.08 | 6(18) | 74 | 0.09 | 10(17) |
|  | *Carnobacterium* | 1 | 0.00 | 1(12) | 57 | 0.08 | 4(18) | 35 | 0.04 | 5(17) |
|  | *Peptococcus* | 1 | 0.00 | 1(12) | 36 | 0.05 | 1(18) | 24 | 0.03 | 3(17) |
|  | *Klebsiella* | 1 | 0.00 | 1(12) | 32 | 0.04 | 2(18) | 9 | 0.01 | 2(17) |
|  | *Acidovorax* | 1 | 0.00 | 1(12) | 21 | 0.03 | 8(18) | 11 | 0.01 | 5(17) |
|  | *Pseudospirillum* | 1 | 0.00 | 1(12) | 17 | 0.02 | 5(18) | 9 | 0.01 | 3(17) |
|  | *Humihabitans* | 1 | 0.00 | 1(12) | 2 | 0.00 | 1(18) | 0 | 0.00 | 0.00 |
|  | *Serratia* | 1 | 0.00 | 1(12) | 1 | 0.00 | 1(18) | 22 | 0.03 | 5(17) |
|  | *Anaeroglobus* | 1 | 0.00 | 1(12) | 1 | 0.00 | 1(18) | 1 | 0.00 | 1(17) |
|  | *Pasteurella* | 1 | 0.00 | 1(12) | 1 | 0.00 | 1(18) | 0 | 0.00 | 0.00 |
|  | *Shuttleworthia* | 1 | 0.00 | 1(12) | 1 | 0.00 | 1(18) | 0 | 0.00 | 0.00 |
|  | *Cardiobacterium* | 1 | 0.00 | 1(12) | 0 | 0.00 | 0.00 | 2 | 0.00 | 2(17) |
|  | *Nitrosomonas* | 1 | 0.00 | 1(12) | 0 | 0.00 | 0.00 | 2 | 0.00 | 1(17) |
|  | *Alysiella* | 1 | 0.00 | 1(12) | 0 | 0.00 | 0.00 | 0 | 0.00 | 0.00 |
|  | *Amycolatopsis* | 1 | 0.00 | 1(12) | 0 | 0.00 | 0.00 | 0 | 0.00 | 0.00 |
|  | *Balneimonas* | 1 | 0.00 | 1(12) | 0 | 0.00 | 0.00 | 0 | 0.00 | 0.00 |
|  | *Blastococcus* | 1 | 0.00 | 1(12) | 0 | 0.00 | 0.00 | 0 | 0.00 | 0.00 |
|  | *Brachybacterium* | 1 | 0.00 | 1(12) | 0 | 0.00 | 0.00 | 0 | 0.00 | 0.00 |
|  | *Candidatus Chloracidobacterium* | 1 | 0.00 | 1(12) | 0 | 0.00 | 0.00 | 0 | 0.00 | 0.00 |
|  | *Cetobacterium* | 1 | 0.00 | 1(12) | 0 | 0.00 | 0.00 | 0 | 0.00 | 0.00 |
|  | *Desulfomicrobium* | 1 | 0.00 | 1(12) | 0 | 0.00 | 0.00 | 0 | 0.00 | 0.00 |
|  | *Flavisolibacter* | 1 | 0.00 | 1(12) | 0 | 0.00 | 0.00 | 0 | 0.00 | 0.00 |
|  | *Flexibacter* | 1 | 0.00 | 1(12) | 0 | 0.00 | 0.00 | 0 | 0.00 | 0.00 |
|  | *Marinobacterium* | 1 | 0.00 | 1(12) | 0 | 0.00 | 0.00 | 0 | 0.00 | 0.00 |
|  | *Marmoricola* | 1 | 0.00 | 1(12) | 0 | 0.00 | 0.00 | 0 | 0.00 | 0.00 |
|  | *Parabacteroides* | 1 | 0.00 | 1(12) | 0 | 0.00 | 0.00 | 0 | 0.00 | 0.00 |
|  | *Pseudidiomarina* | 1 | 0.00 | 1(12) | 0 | 0.00 | 0.00 | 0 | 0.00 | 0.00 |
|  | *Solirubrobacter* | 1 | 0.00 | 1(12) | 0 | 0.00 | 0.00 | 0 | 0.00 | 0.00 |
|  | *Thermomonas* | 1 | 0.00 | 1(12) | 0 | 0.00 | 0.00 | 0 | 0.00 | 0.00 |
|  | *Virgibacillus* | 1 | 0.00 | 1(12) | 0 | 0.00 | 0.00 | 0 | 0.00 | 0.00 |
|  | *Citrobacter* | 0 | 0.00 | 0.00 | 1319 | 1.77 | 3(18) | 10.00 | 0.01 | 3(17) |
|  | *Leuconostoc* | 0 | 0.00 | 0.00 | 901 | 1.21 | 4(18) | 1090 | 1.39 | 6(17) |
|  | *Raoultella* | 0 | 0.00 | 0.00 | 230 | 0.31 | 3(18) | 13 | 0.02 | 1(17) |
|  | *Clostridium* | 0 | 0.00 | 0.00 | 79 | 0.11 | 2(18) | 2.00 | 0.00 | 2(17) |
|  | *Salmonella* | 0 | 0.00 | 0.00 | 73 | 0.10 | 1(18) | 0.00 | 0.00 | 0.00 |
|  | *Pantoea* | 0 | 0.00 | 0.00 | 28 | 0.04 | 1(18) | 2.00 | 0.00 | 1(17) |
|  | *Cronobacter* | 0 | 0.00 | 0.00 | 27 | 0.04 | 2(18) | 0.00 | 0.00 | 0.00 |
|  | *Tatumella* | 0 | 0.00 | 0.00 | 25 | 0.03 | 7(18) | 8.00 | 0.01 | 5(17) |
|  | *Escherichia* | 0 | 0.00 | 0.00 | 10 | 0.01 | 1(18) | 2.00 | 0.00 | 1(17) |
|  | *Xenorhabdus* | 0 | 0.00 | 0.00 | 9 | 0.01 | 2(18) | 2.00 | 0.00 | 2(17) |
|  | *Alteromonas* | 0 | 0.00 | 0.00 | 9 | 0.01 | 5(18) | 1.00 | 0.00 | 1(17) |
|  | *Sinobaca* | 0 | 0.00 | 0.00 | 6 | 0.01 | 2(18) | 9.00 | 0.01 | 5(17) |
|  | *Azotobacter* | 0 | 0.00 | 0.00 | 6 | 0.01 | 4(18) | 2.00 | 0.00 | 1(17) |
|  | *Weissella* | 0 | 0.00 | 0.00 | 5 | 0.01 | 2(18) | 10.00 | 0.01 | 2(17) |
|  | *Shewanella* | 0 | 0.00 | 0.00 | 4 | 0.01 | 2(18) | 5.00 | 0.01 | 2(17) |
|  | *Planomicrobium* | 0 | 0.00 | 0.00 | 4 | 0.01 | 2(18) | 1.00 | 0.00 | 1(17) |
|  | *Enterococcus* | 0 | 0.00 | 0.00 | 3 | 0.00 | 1(18) | 7.00 | 0.01 | 3(17) |
|  | *Globicatella* | 0 | 0.00 | 0.00 | 3 | 0.00 | 1(18) | 2.00 | 0.00 | 1(17) |
|  | *Roseiflexus* | 0 | 0.00 | 0.00 | 3 | 0.00 | 2(18) | 2.00 | 0.00 | 1(17) |
|  | *Cedecea* | 0 | 0.00 | 0.00 | 3 | 0.00 | 1(18) | 0.00 | 0.00 | 0.00 |
|  | *Bradyrhizobium* | 0 | 0.00 | 0.00 | 2 | 0.00 | 2(18) | 129 | 0.16 | 2(17) |
|  | *Kluyvera* | 0 | 0.00 | 0.00 | 2 | 0.00 | 1(18) | 17 | 0.02 | 2(17) |
|  | *Lysinibacillus* | 0 | 0.00 | 0.00 | 2 | 0.00 | 2(18) | 3.00 | 0.00 | 1(17) |
|  | *Candidatus contubernalis* | 0 | 0.00 | 0.00 | 2 | 0.00 | 1(18) | 0.00 | 0.00 | 0.00 |
|  | *Macrococcus* | 0 | 0.00 | 0.00 | 2 | 0.00 | 1(18) | 0.00 | 0.00 | 0.00 |
|  | *Micrococcus* | 0 | 0.00 | 0.00 | 2 | 0.00 | 1(18) | 0.00 | 0.00 | 0.00 |
|  | *Flavobacterium* | 0 | 0.00 | 0.00 | 1 | 0.00 | 1(18) | 2431 | 3.10 | 1(17) |
|  | *Rahnella* | 0 | 0.00 | 0.00 | 1 | 0.00 | 1(18) | 100 | 0.13 | 3(17) |
|  | *Ignavigranum* | 0 | 0.00 | 0.00 | 1 | 0.00 | 1(18) | 6.00 | 0.01 | 1(17) |
|  | *Yersinia* | 0 | 0.00 | 0.00 | 1 | 0.00 | 1(18) | 6.00 | 0.01 | 2(17) |
|  | *Zimmermannella* | 0 | 0.00 | 0.00 | 1 | 0.00 | 1(18) | 5.00 | 0.01 | 2(17) |
|  | *Facklamia* | 0 | 0.00 | 0.00 | 1 | 0.00 | 1(18) | 3.00 | 0.00 | 1(17) |
|  | *Pectobacterium* | 0 | 0.00 | 0.00 | 1 | 0.00 | 1(18) | 2.00 | 0.00 | 2(17) |
|  | *Helicobacter* | 0 | 0.00 | 0.00 | 1 | 0.00 | 1(18) | 1.00 | 0.00 | 1(17) |
|  | *Rathayibacter* | 0 | 0.00 | 0.00 | 1 | 0.00 | 1(18) | 1.00 | 0.00 | 1(17) |
|  | *Achromobacter* | 0 | 0.00 | 0.00 | 1 | 0.00 | 1(18) | 0.00 | 0.00 | 0.00 |
|  | *Bdellovibrio* | 0 | 0.00 | 0.00 | 1 | 0.00 | 1(18) | 0.00 | 0.00 | 0.00 |
|  | *Caenispirillum* | 0 | 0.00 | 0.00 | 1 | 0.00 | 1(18) | 0.00 | 0.00 | 0.00 |
|  | *Diaphorobacter* | 0 | 0.00 | 0.00 | 1 | 0.00 | 1(18) | 0.00 | 0.00 | 0.00 |
|  | *Erysipelothrix* | 0 | 0.00 | 0.00 | 1 | 0.00 | 1(18) | 0.00 | 0.00 | 0.00 |
|  | *Eubacterium* | 0 | 0.00 | 0.00 | 1 | 0.00 | 1(18) | 0.00 | 0.00 | 0.00 |
|  | *Ferrithrix* | 0 | 0.00 | 0.00 | 1 | 0.00 | 1(18) | 0.00 | 0.00 | 0.00 |
|  | *Glaciecola* | 0 | 0.00 | 0.00 | 1 | 0.00 | 1(18) | 0.00 | 0.00 | 0.00 |
|  | *Gordonia* | 0 | 0.00 | 0.00 | 1 | 0.00 | 1(18) | 0.00 | 0.00 | 0.00 |
|  | *Hydrogenophaga* | 0 | 0.00 | 0.00 | 1 | 0.00 | 1(18) | 0.00 | 0.00 | 0.00 |
|  | *Methylobacterium* | 0 | 0.00 | 0.00 | 1 | 0.00 | 1(18) | 0.00 | 0.00 | 0.00 |
|  | *Oceanospirillum* | 0 | 0.00 | 0.00 | 1 | 0.00 | 1(18) | 0.00 | 0.00 | 0.00 |
|  | *Pelagibius* | 0 | 0.00 | 0.00 | 1 | 0.00 | 1(18) | 0.00 | 0.00 | 0.00 |
|  | *Pseudobutyrivibrio* | 0 | 0.00 | 0.00 | 1 | 0.00 | 1(18) | 0.00 | 0.00 | 0.00 |
|  | *Sideroxydans* | 0 | 0.00 | 0.00 | 1 | 0.00 | 1(18) | 0.00 | 0.00 | 0.00 |
|  | *Slackia* | 0 | 0.00 | 0.00 | 1 | 0.00 | 1(18) | 0.00 | 0.00 | 0.00 |
|  | *Sulfobacillus* | 0 | 0.00 | 0.00 | 1 | 0.00 | 1(18) | 0.00 | 0.00 | 0.00 |
|  | *Trichococcus* | 0 | 0.00 | 0.00 | 1 | 0.00 | 1(18) | 0.00 | 0.00 | 0.00 |
|  | *Janthinobacterium* | 0 | 0.00 | 0.00 | 0 | 0.00 | 0.00 | 114 | 0.15 | 1(17) |
|  | *Psychrobacter* | 0 | 0.00 | 0.00 | 0 | 0.00 | 0.00 | 9.00 | 0.01 | 9(17) |
|  | *Chryseobacterium* | 0 | 0.00 | 0.00 | 0 | 0.00 | 0.00 | 3.00 | 0.00 | 1(17) |
|  | *Aquimarina* | 0 | 0.00 | 0.00 | 0 | 0.00 | 0.00 | 2.00 | 0.00 | 1(17) |
|  | *Delftia* | 0 | 0.00 | 0.00 | 0 | 0.00 | 0.00 | 2.00 | 0.00 | 1(17) |
|  | *Dokdonella* | 0 | 0.00 | 0.00 | 0 | 0.00 | 0.00 | 2.00 | 0.00 | 1(17) |
|  | *Ferritrophicum* | 0 | 0.00 | 0.00 | 0 | 0.00 | 0.00 | 2.00 | 0.00 | 1(17) |
|  | *Marinomonas* | 0 | 0.00 | 0.00 | 0 | 0.00 | 0.00 | 2.00 | 0.00 | 1(17) |
|  | *Pseudoalteromonas* | 0 | 0.00 | 0.00 | 0 | 0.00 | 0.00 | 2.00 | 0.00 | 1(17) |
|  | *Acholeplasma* | 0 | 0.00 | 0.00 | 0 | 0.00 | 0.00 | 1.00 | 0.00 | 1(17) |
|  | *Alcanivorax* | 0 | 0.00 | 0.00 | 0 | 0.00 | 0.00 | 1.00 | 0.00 | 1(17) |
|  | *Arthrobacter* | 0 | 0.00 | 0.00 | 0 | 0.00 | 0.00 | 1.00 | 0.00 | 1(17) |
|  | *Colwellia* | 0 | 0.00 | 0.00 | 0 | 0.00 | 0.00 | 1.00 | 0.00 | 1(17) |
|  | *Desulfovibrio* | 0 | 0.00 | 0.00 | 0 | 0.00 | 0.00 | 1.00 | 0.00 | 1(17) |
|  | *Edwardsiella* | 0 | 0.00 | 0.00 | 0 | 0.00 | 0.00 | 1.00 | 0.00 | 1(17) |
|  | *Gelria* | 0 | 0.00 | 0.00 | 0 | 0.00 | 0.00 | 1.00 | 0.00 | 1(17) |
|  | *Geobacillus* | 0 | 0.00 | 0.00 | 0 | 0.00 | 0.00 | 1.00 | 0.00 | 1(17) |
|  | *Massilia* | 0 | 0.00 | 0.00 | 0 | 0.00 | 0.00 | 1.00 | 0.00 | 1(17) |
|  | *Mobiluncus* | 0 | 0.00 | 0.00 | 0 | 0.00 | 0.00 | 1.00 | 0.00 | 1(17) |
|  | *Naxibacter* | 0 | 0.00 | 0.00 | 0 | 0.00 | 0.00 | 1.00 | 0.00 | 1(17) |
|  | *Nitrincola* | 0 | 0.00 | 0.00 | 0 | 0.00 | 0.00 | 1.00 | 0.00 | 1(17) |
|  | *Prauserella* | 0 | 0.00 | 0.00 | 0 | 0.00 | 0.00 | 1.00 | 0.00 | 1(17) |
|  | *Rhodococcus* | 0 | 0.00 | 0.00 | 0 | 0.00 | 0.00 | 1.00 | 0.00 | 1(17) |
|  | *Sarcina* | 0 | 0.00 | 0.00 | 0 | 0.00 | 0.00 | 1.00 | 0.00 | 1(17) |
|  | *Solibacillus* | 0 | 0.00 | 0.00 | 0 | 0.00 | 0.00 | 1.00 | 0.00 | 1(17) |
|  | *Sphingobium* | 0 | 0.00 | 0.00 | 0 | 0.00 | 0.00 | 1.00 | 0.00 | 1(17) |
|  | *Streptobacillus* | 0 | 0.00 | 0.00 | 0 | 0.00 | 0.00 | 1.00 | 0.00 | 1(17) |
|  | *Tissierella* | 0 | 0.00 | 0.00 | 0 | 0.00 | 0.00 | 1.00 | 0.00 | 1(17) |
|  | *Williamsia* | 0 | 0.00 | 0.00 | 0 | 0.00 | 0.00 | 1.00 | 0.00 | 1(17) |
